# In pain and lonely? A longitudinal study examining the associations between menstrual pain, physical functioning and loneliness

**DOI:** 10.1111/bjhp.12805

**Published:** 2025-05-26

**Authors:** J. McCurry, D. Skvarc, S. Evans, A. Mikocka‐Walus, M. L. Druitt, L. Payne, E. M. Marshall

**Affiliations:** ^1^ School of Psychology Deakin University Geelong Victoria Australia; ^2^ University Hospital Geelong Geelong Victoria Australia; ^3^ School of Medicine Deakin University Geelong Victoria Australia; ^4^ Division of Women's Mental Health McLean Hospital Belmont Massachusetts USA; ^5^ Harvard Medical School Boston Massachusetts USA

**Keywords:** chronic pain, dysmenorrhea, loneliness, pain severity, physical functioning

## Abstract

**Background and Aims:**

The current study aimed to determine the prospective associations between menstrual pain, physical health functioning and loneliness.

**Method:**

We collected three waves of data from a community cohort of women reporting at least mild menstrual pain over 3 years. Participants were women aged 18–50, living in Australia, who had experienced regular menses and menstrual pain in the past year. Two hundred and eighty‐nine women (*n* = 100 with self‐reported endometriosis, 34% of current sample) completed the online questionnaires at all three time points and had complete data assessing pain severity, physical functioning and loneliness.

**Results:**

Poorer physical functioning was associated with greater loneliness (*β* = −.371, *p* < .001). Contrary to our expectations, menstrual pain severity was not associated directly with loneliness but was indirectly associated via physical functioning (*R* ^2^ = .195, *p* < 001).

**Discussion:**

The overall stability in physical functioning over time suggests that a person‘s degree of physical functioning could be an early indicator of loneliness experiences. Health professionals wanting to mitigate loneliness amongst people with menstrual pain might benefit from screening for and addressing physical functioning impairment. It might also be beneficial to screen for and treat menstrual pain to ensure that it does not lead to or exacerbate physical functioning impairment.

**Conclusion:**

Future research should consider the underlying mechanisms that drive the associations between physical functioning and loneliness.

## INTRODUCTION

Pain is common during the menstrual cycle. However, the severity of this pain varies considerably. In one study of thousand fifty one 15‐ to 19‐year‐old girls, 93% reported some degree of menstrual pain with 31% reporting no‐to‐mild pain, 48% reporting moderate pain and 21% reporting severe pain (Parker et al., 2010). The experience of moderate‐to‐severe menstrual pain is referred to as dysmenorrhea, and it is estimated to affect ~80% of menstruating people, making it one of the most common menstrual symptom (Armour et al., 2022; Schoep et al., 2019; Subasinghe et al., 2016). Dysmenorrhea can occur with or without visible pelvic pathology, such as endometriosis (a chronic health condition where endometrial tissue appears outside the uterus; Proctor & Farquhar, 2006). Of concern, dysmenorrhea has a significant and widespread impact on a person and the broader community (Culley et al., 2013; Evans et al., 2019; Iacovides et al., 2015), resulting in a significant economic burden estimated at around $A6.5 billion per annum (Armour et al., 2022; Australian Government Department of Health, 2018; MacGregor et al., 2023). Despite the prevalence and widespread economic and personal impact of menstrual pain, the social implications associated with it remain understudied and poorly understood (Gagnon et al., 2023). This is concerning given that menstrual health is a critical aspect of health, human rights and gender equity (World Health Organization, 2022), and is defined to be—‘a state of complete physical, mental and *social* wellbeing in relation to the menstrual health’ (Hennegan et al., 2021, p. 32).

One assumed social implication of menstrual pain is the experience of loneliness (Allyn et al., 2020; Chandel et al., 2023; Cole et al., 2021), which extends beyond the objective experience of social isolation to capture the subjective experience of feeling alone (Cacioppo et al., 2006; Weiss, 1973). This subjective experience of loneliness arises when a person perceives discrepancies between their actual and preferred levels of social connection (Perlman & Peplau, 1981). Such discrepancies relate to the structure of a person's social network (e.g. the number of relationships and the frequency of social contact) or the functions of a person's social relationships (e.g. the intimacy or quality of the relationships and the support that they provide) (Dahlberg, 2021). Loneliness can also arise from an absence or want for close relationships (emotional loneliness) or due to the absence or want for social connection more broadly (social loneliness) (Weiss, 1973).

While qualitative research asserts that loneliness occurs when women experience moderate–severe pain (Cole et al., 2021; Peterson et al., 2023), quantitative research has not systematically examined whether this is the case. Of particular importance, no research to our knowledge has been able to determine whether loneliness is associated with the experience of menstrual pain alone or whether loneliness is more strongly associated with any co‐occurring impairment of physical functioning (Iacovides et al., 2015; MacGregor et al., 2023). Since physical impairment often presents with external features, impairments may be more reliably screened for during routine check‐ups and assessments, assisting clinicians to screen for and identify those at risk to ensure that they experience optimum levels of menstrual health. Thus, our current 3‐wave study aims to advance our understanding of menstrual health by understanding the associations between menstrual pain, physical functioning and loneliness using a sample of women who experience some degree of menstrual pain.

### Loneliness and menstrual pain

There is strong evidence to suggest that loneliness has significant adverse mental and physical health outcomes (Hawkley et al., 2022). A comprehensive meta‐analysis by Holt‐Lunstad et al. (2015) found that loneliness is associated with a 26% increased risk of premature mortality, a 32% increased risk of stroke and a 29% increased risk of developing cardiovascular disease. Research further indicates that loneliness is associated with depression (Cacioppo et al., 2006), substance misuse (Dyal & Valente, 2015), high blood pressure (Momtaz et al., 2012) and metabolic syndrome (Whisman, 2010). As such, loneliness has become a significant public health concern over the last decade (Lim et al., 2020), with the United Kingdom appointing a Minister for Loneliness (U.K. Department for Digital, Culture, Media and Sport, 2022) and the United States Surgeon General declaring a ‘loneliness epidemic’ (Bruce et al., 2019). The COVID‐19 pandemic exacerbated the problem further (Ernst et al., 2022).

While loneliness is a common experience in the general population (Holt‐Lunstad, 2017), loneliness is even more pronounced in people with pain conditions (Richard et al., 2017), including fibromyalgia (Wolf et al., 2015) and rheumatoid arthritis (El‐Mansoury et al., 2008). Indeed, longitudinal evidence suggests that physical pain in the bones, joints and muscles is associated with increased feelings of loneliness and that this association occurs even when accounting for social isolation (Boggero et al., 2019; Smith et al., 2019). Moreover, longitudinal research into chronic inflammatory pain found that participants with severe pain were seven times more likely to experience higher levels of loneliness after 4 years when compared with participants experiencing only mild and moderate pain (Loeffler & Steptoe, 2021).

It is clear from the dysmenorrhea literature that women with increased menstrual pain have a poorer quality of life, which includes poorer social functioning (Iacovides et al., 2015; MacGregor et al., 2023). Indeed, research has shown that women often withdraw socially and professionally when they experience menstrual pain (Allyn et al., 2020; Culley et al., 2013; Mills et al., 2025; Peterson et al., 2023; Serrahima & Martínez, 2023) and a significant percentage is unable to undertake daily activities when menstruating (Schoep et al., 2019). Concerningly, such withdrawal may begin early in life, with some estimates suggesting that three‐quarters of a student sample practised self‐isolation due to menstrual pain (MacGregor et al., 2023; Yesuf et al., 2018). Qualitative research also suggests that experiences of loneliness are a common for women with menstrual pain (Chandel et al., 2023; Cole et al., 2021; Peterson et al., 2023).

**FIGURE 1:**
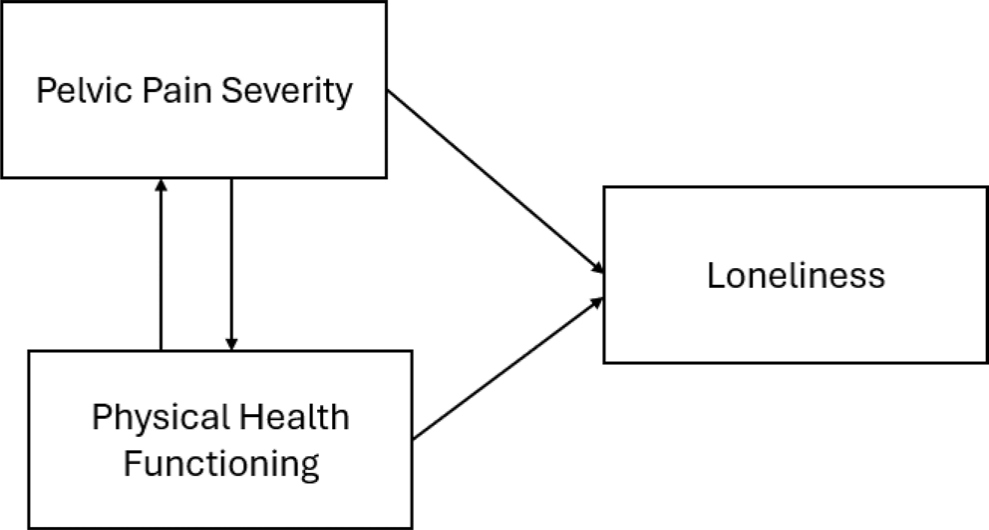
The theorized associations between pain severity, physical health functioning and loneliness.

It is clear that menstrual pain, particularly moderate‐to‐severe menstrual pain (i.e. dysmenorrhea), has significant social implications (see also Iacovides et al., 2015; MacGregor et al., 2023), and there is evidence to suggest that this extends to the experience of loneliness more specifically (Chandel et al., 2023; Cole et al., 2021; Peterson et al., 2023). However, we question whether menstrual pain is directly associated with experiences of loneliness or whether loneliness is better explained by impairment of physical functioning, or a reduced ability to perform basic and desired activities of daily living that often co‐occurs or arises from the pain (Dias et al., 2021; Iacovides et al., 2015; MacGregor et al., 2023) (see Figure 1). Dysmenorrhea severity is often associated with lower levels of physical functioning, regardless of the woman's age (Bala et al., 2024; Fourquet et al., 2010; Iacovides et al., 2015; MacGregor et al., 2023). In addition to impaired mobility, women described being frequently woken up from sleep due to uterine cramps (Allyn et al., 2020) and experiencing daytime dysfunction (i.e. low energy, fatigue, sleepiness; Cole et al., 2021). However, there is also evidence to show that the impact of menstrual pain is not uniform, with some experiencing more impairment than others (MacGregor et al., 2023). While there is qualitative evidence to suggest that the impairment of physical functioning is what underlies and drives the assumed association between menstrual pain and social wellbeing (Peterson et al., 2023), to our knowledge, no research has examined these relationships using quantitative data.

### The current study

This prospective study uses data obtained at three time points from a community cohort of women with some degree of menstrual pain to examine the associations between pain severity and physical functioning over time and the extent to which these factors are both associated with perceptions of loneliness. More specifically, we examine the process outlined in Figure 1 by examining the bidirectional associations between menstrual pain severity and physical health functioning over the three time points and the extent to which these factors predict loneliness at Time 3. We hypothesized that higher levels of menstrual pain severity and lower levels of physical functioning would be associated with higher levels of loneliness in menstruating women aged between 18 and 50 years old. However, we expected physical functioning to be more strongly associated with loneliness.

## METHOD

### Procedure and participants

The current study is related to a larger longitudinal cohort study that examined menstrual health and wellbeing in a sample of Australian women aged 18–50 years of age (Evans et al., 2021). The larger study collected baseline data at Time 1 (T1; May 2019), and we collected data at two additional time points: Time 2 (T2; June 2020) and Time 3 (T3; June 2021). Data from this study included all three time points. Institutional ethics approval was granted prior to data collection. We recruited participants via social media (including gyms, peer support groups and university forums). We directed interested participants to a web‐based survey created using Qualtrics. Participants indicated their consent to participate, were advised of the study's purpose and were assured that their participation was anonymous, voluntary and confidential. Participants who completed the baseline survey and consented to the follow‐ups were sent a survey link via email in June 2020 and June 2021.

Our final sample included those with complete data over the three time points and those whose data could be estimated through maximum likelihood (*N* = 289 women; *M*_*age*_ = 28.92, SD = 7.48). We included women aged between 18 and 50 years, living in Australia, who had experienced regular periods in the past year and experienced at least mild menstrual pain in the past year. The sample consisted of participants who were primarily born in Australia (78.9%), employed full or part time (59.9%) and not in a relationship (52.9%), which corresponded well with the Australian population (ABS, 2021). Around two‐thirds of participants reported not having endometriosis (65.4%), and over one‐third reported having one or more chronic illnesses (37.7%; see Table 1 for detailed demographics).

**TABLE 1:** Descriptive and participant demographic statistics.

	Mean	SD	Min	Max	*n*
Age	28.92	7.48	18	50	289
Pain level (T1)	6.53	2.24	1	10	
Pain level (T2)	6.31	2.11	1	10	
Pain level (T3)	6.19	1.98	1	10	
Physical QoL (T1)	22.98	5.1	8	35	
Physical QoL (T2)	23.72	5.34	10	35	
Physical QoL (T3)	23.02	5.35	7	34	
Loneliness (T3)	7.83	3.09	4	16	

	*N*	%
Marital status		
Single (never married)	138	47.80
Married/de facto	136	47.10
Widowed	1	0.30
Divorced	8	2.80
Separated	1	0.30
Other (please specify)	5	1.70
Employment status		
Employed full time	106	36.70
Employed part time	67	23.20
Unemployed and currently looking for work	7	2.40
Unemployed and not currently looking for work	1	0.30
Student	71	24.60
Retired	0	0.00
Homemaker	13	4.50
Self‐employed	7	2.40
Unable to work	10	3.50
Other (please specify)	7	2.40
Endometriosis diagnosis		
Yes	100	34.60
No	189	65.40
Chronic Illness diagnosis		
Yes	109	37.70
No	180	62.30
Home language		
English	251	96.90
Other (please specify)	8	3.10
Birth country		
Australia	228	78.9
Elsewhere	61	21.1
Australian Aboriginal or Torres Strait Islander		
Yes	7	2.70
No	252	97.30

Abbreviation: Physical QoL, Physical Functioning.

### Measures

#### Demographics and covariates (T1 only)

To account for expected covariates of menstrual pain and loneliness, we recorded age, employment status, marital status, number of children, endometriosis status and other chronic illness status.

#### Loneliness (T3 only)

The Short‐Form UCLA Loneliness Scale (UCLA‐4; Russell et al., 1980) is a 4‐item, 4‐point (1 = never to 4 = always) Likert‐type scale, measuring loneliness. Item examples are ‘I lack companionship’ and ‘I feel left out’. Relevant items were reversed scored, and scores were summed to compute a total score (possible range = 4–16). Higher scores indicated greater loneliness. The UCLA‐4 has demonstrated concurrent validity and has demonstrated excellent reliability (*α* = .90; Russell, 1996). The current study demonstrated good internal consistency (*α* = .85). Average loneliness scores for adults are around 5.5/16 (Hays & DiMatteo, 1987).

#### Menstrual pain severity (T1, T2 and T3)

A numeric rating scale (NRS; Chen et al., 2015) was used to assess the severity of menstrual pain by asking participants to rate their ‘usual level of period pain (without medication)’ on an 11‐point interval scale (0 = *no pain to* 10 = *worst pain possible*). Dysmenorrhea is defined as having moderate‐to‐severe menstrual pain, as evidenced by an NRS rating of at least 4 (possible range 1–10; Chen et al., 2015). We were interested in understanding the impact of even mild menstrual pain, so we included all participants with a score of at least one. The NRS has demonstrated good construct validity and has been extensively used in women with menstrual pain (Hawker et al., 2011; Young et al., 2015).

#### Physical functioning (T1, T2 and T3)

The World Health Organization Quality of Life Assessment‐Brief (WHOQOL‐BREF; The WHOQOL Group, 1998) is a 26‐item, 5‐point (1 = *not at all/very dissatisfied* to 5 = *completely/very satisfied*) Likert‐type scale measuring quality of life satisfaction. The WHOQOL‐BREF comprises four subscales: physical, social, psychological and environmental. The current study used the 7‐item physical health subscale. Example items include ‘to what extent do you feel that physical pain prevents you from doing what you need to do?’ and ‘how well are you able to get around?’. Relevant items were reversed scored, and scores were summed to compute a total score (possible range = 7–35). Higher scores indicated greater physical functioning satisfaction. The WHOQOL‐BREF has demonstrated good concurrent validity for the physical health domain and is strongly correlated with the physical function, physical roles, bodily pain, general health, vitality and total physical score subscales on the Short‐Form Health Survey (Skevington, 2010), and with the physical and independence domains of the Subjective Quality of Life Profile (Bonomi et al., 2000). The current study demonstrated good internal consistency at T1 (*α* = .82), T2 (*α* = .84) and T3 (*α* = .85).

### Data analysis

We conducted descriptive statistics in IBM SPSS 29 and the primary analyses in AMOS version 29. Missing data were MCAR and so were estimated using maximum likelihood. We compared participants who dropped out of the study to those participants who completed all time points. Overall, we observed no significant differences except for the inability to work. Significantly more participants who dropped out could not work at baseline (see the Data S1). We observed no evidence of non‐normality through examination of standardized residuals, and so, we performed no data transformation.

**FIGURE 2:**
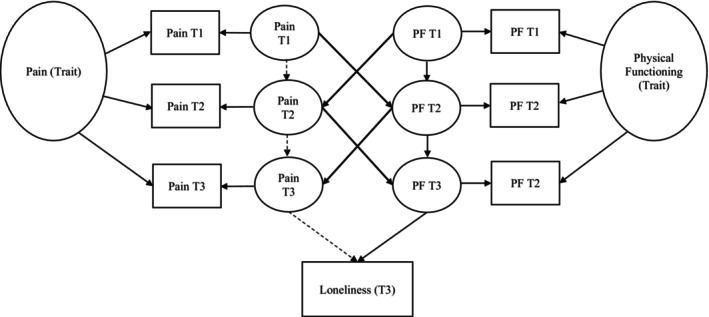
The RI‐CPLM model used to examine the associations between the predictors (pain severity and physical functioning, PF) and the outcome (loneliness), while modelling the within‐person variations and stable‐trait‐like aspects of the predictors. The dashed lines indicate the non‐significant paths, and the full lines indicate the significant paths. The model also included endometriosis diagnosis, other chronic illnesses, employment, age and marital status as confounders. For interpretation, the covariations and errors are not included in the current figure. The complete figure is included in the Data S1. Pain, pain severity; PF, physical functioning; T1/T2/T3, Time 1, Time 2 & Time 3.

For our primary analyses, we constructed a three‐wave autoregressive, random intercepts cross‐panel longitudinal model (RI‐CLPM; Hamaker et al., 2015; Lucas, 2023). The RI‐CLPM used in the current study (see Figure 2) accounts for the presence of within‐person and between‐person variation in pain and physical functioning and minimizes the risk of spurious effects without overly increasing the Type II error rate (Hamaker et al., 2015; Lucas, 2023). More specifically, the RI‐CLPM includes latent pain and physical functioning variables, which follow an autoregressive structure. Thus, each person's prior report of pain (or physical functioning) (Time‐1) predicts their following report of pain (or physical functioning) (Time‐2). We also included cross‐lagged associations where each person's prior report of pain (or physical functioning; Time‐1) predicts their next report of physical functioning (or pain; Time‐2). In addition, the RI‐CLPM uses correlated latent stable‐trait ratings of pain and physical functioning to predict participant loneliness at T3. The model reported in our main analyses includes potential confounding variables: age, endometriosis status, marital status, employment and chronic illness status (refer to the Data S1 for the model's output). The results of the primary research aim did not change between adjusted and unadjusted models.

## RESULTS

### Descriptive statistics and bivariate correlations

The descriptive statistics and bivariate correlations for the study variables are reported in Tables 1 and 2. On average, participants reported moderate levels of menstrual pain and impairment of physical functioning throughout the study and average levels of loneliness. We observed significant bivariate correlations between all variables in the unadjusted model except for time one pain and loneliness (Time‐3).

**TABLE 2:** Bivariate correlations between the study variables.

Variable		1	2	3	4	5	6
1. Pain level (T1)	*r*						
	*p*						
	*n*						
2. Pain level (T2)	*r*	.679***					
	*p*	<.001					
	*n*	502					
3. Pain level (T3)	*r*	.602***	.634***				
	*p*	<.001	<.001				
	*n*	326	241				
4. Physical function (T1)	*r*	−.392***	−.325***	−.178*			
	*p*	<.001	<.001	.001			
	*n*	1540	501	326			
5. Physical function (T2)	*r*	−.353***	−.397***	−.286***	.725***		
	*p*	<.001	<.001	<.001	<.001		
	*n*	465	465	226	464		
6. Physical function (T3)	*r*	−.249***	−.298***	−.319***	.654***	.709***	
	*p*	<.001	<.001	<.001	<.001	<.001	
	*n*	317	232	317	317	220	
7. Loneliness (T3)	*r*	0.109	.162*	.130*	−.315***	−.265***	−.377***
	*p*	0.064	0.017	0.027	<.001	<.001	<.001
	*n*	289	217	289	289	207	289

*Note*: *r*, *p* and *n* refer to the bivariate correlation, *p* value and analysis cases per correlation. *, ** and *** indicate statistical significance at .05, .01 and .001 levels.

### Main analyses

We examined model fit with the Tucker‐Lewis Fit Index (TLI), Comparative Fit Index (CFI; acceptable fit indicated by scores of .950) and Root Mean Square Error of Approximation (RMSEA, <.05). The model estimated 72 parameters and retained 32 degrees of freedom (x^2^ = 69.91, *p* < .001). The specified model achieved excellent fit: TLI = 0.970, CFI = 0.990, RMSEA = .022.

The parameters of the RI‐CLPM pertaining to the research aim are reported in Table 3. All other results (i.e. covariances and correlations between latent stable traits) are reported in the Data S1. As shown, the autoregressive pathways suggest overall stability for physical functioning but not pain level over time. All cross‐lagged pathways were significant, suggesting that pain level and physical functioning reciprocally predicted each other over time. Further, significant negative associations were found between pain level at times one and two and physical functioning at times two and three, and the inverse was also true. We observed no significant relationship between pain at time three and loneliness but observed a significant and negative association between time three physical functioning and loneliness. The results, therefore, suggest that higher physical functioning (i.e. lower physical impairment) predicts lower levels of loneliness in women with menstrual pain, even after controlling for the severity of pain and other covariates. Our significant cross‐lagged effects also indicate the presence of indirect effects, which, coupled with the significant association between physical function at Time‐3 and the lack of a comparable association with pain at Time‐3, suggest that physical functioning substantially accounts for the relationship between pain and loneliness. In other words, physical dysfunction secondary to pain is the primary, though indirect, driver of loneliness in our model. The overall stability in physical functioning suggests its value as an early indicator for future loneliness experiences.

**TABLE 3:** Regression pathway parameters for the RI‐CLPM model.

Exogenous (predictor)	Endogenous (outcome)	Estimate	*β*	SE	CR	*p*
Pain (T1)	Pain (T2)	0.19	0.18	0.098	1.928	0.054
Pain (T2)	Pain (T3)	0.19	0.20	0.098	1.928	0.054
Pain (T1)*	Physical functioning (T2)	−0.618	−0.216	0.225	−2.752	0.006
Physical functioning (T1)***	Physical functioning (T2)	0.465	0.442	0.085	5.442	<.001
Physical functioning (T2)***	Physical functioning (T3)	0.465	0.463	0.085	5.442	<.001
Physical functioning (T1)***	Pain (T2)	−0.135	−0.353	0.033	−4.096	<.001
Physical functioning (T2)**	Pain (T3)	−0.113	−0.329	0.034	−3.279	0.001
Pain (T2)**	Physical functioning (T3)	−0.576	−0.209	0.208	−2.776	0.005
Pain (T3)	Loneliness (T3)	0.056	0.027	0.166	0.338	0.735
Physical functioning (T3)***	Loneliness (T3)	−0.267	−0.371	0.058	−4.562	<.001
Endometriosis	Loneliness (T3)	0.37	0.058	0.358	1.035	0.301
Chronic illness**	Loneliness (T3)	−1.007	−0.157	0.359	−2.803	0.005
Employment status	Loneliness (T3)	0.131	0.108	0.068	1.93	0.054
Age	Loneliness (T3)	−0.039	−0.099	0.028	−1.412	0.158
Marital status	Loneliness (T3)	0.138	0.039	0.212	0.648	0.517
Number of children	Loneliness (T3)	−0.103	−0.015	0.474	−0.216	0.829

Endogenous (outcome)	*R* ^2^	LL95	UL95
Physical functioning (T2)	0.344	0.312	0.375
Pain (T2)	0.226	0.196	0.255
Physical functioning (T3)	0.364	0.338	0.394
Pain (T3)	0.222	0.192	0.251
Loneliness (T3)	0.195	0.166	0.223

*Note*: *, ** and *** indicate statistical significance at .05, .01 and .001 levels.

Abbreviations: CR, critical ratio; LL95 & UL95, lower and upper limit, 95% confidence interval.

## DISCUSSION

The current study examined the associations between menstrual pain severity, physical functioning and loneliness over time in a community cohort of women. As hypothesized, a lower level of physical functioning was significantly associated with higher levels of loneliness even when controlling for age, employment status, marital status, number of children, endometriosis status and other chronic illnesses. The overall stability of physical functioning over time suggests that it is not only a concurrent predictor of loneliness but also a prospective predictor of loneliness. Contrary to our expectations, however, menstrual pain severity was not directly associated with loneliness and only indirectly associated with loneliness via physical functioning, constituting a partial mediation effect. More specifically, and consistent with common biopsychosocial models of pain (Carr, 2011), higher pain was associated with lower physical functioning, which in turn was associated with higher loneliness. While our findings are preliminary, they indicate that screening menstruating women (or menstruating people) for impairment of physical functioning might assist health professionals in identifying vulnerable women for loneliness interventions. It might also be beneficial for loneliness interventions to improve physical functioning actively or, at the very least, ensure that it does not interfere with social connections and social functioning. It is also worth tracking women with high pain levels to offset any future impairment of physical functioning and heightened loneliness experiences, and the physical functioning scale of the WHOQOL‐BREF (which is globally validated and available in many languages) would be helpful for this purpose, though a direct inquiry from the clinician to the patient could also suffice.

The negative concurrent association found between physical functioning and loneliness is consistent with previous findings that examined participants with rheumatoid arthritis (El‐Mansoury et al., 2008) and fibromyalgia (Smith et al., 2019), two pain conditions that have features overlapping with dysmenorrhea (i.e. chronic pain with acute pain flare‐ups; Greenbaum et al., 2019). Research indicates that physical functioning restrictions interfere with multiple aspects of people's social lives, including the ability to attend work and engage in social, family and leisure activities (Garber et al., 2010; Gyasi et al., 2022; Rowlands et al., 2022). Evidence suggests that people with worse mobility impairment are less likely to engage with their community (Hawkley & Cacioppo, 2010; Smith et al., 2019), which can impair a broader sense of belonging, therefore leading to an increased risk of loneliness (Michalski et al., 2020). Impairment of physical functioning can also reduce one's ability to work since people may need to take sick leave or be unable to fulfil the job requirement (i.e. manual labour, standing for long periods; Armour et al., 2022). Likewise, physical impairments hamper participation in sports and recreation, so those affected are less likely to foster social networks through these avenues and contribute to feeling different to others (Iovino et al., 2023). It is also worth noting that the experience of moderate–severe menstrual pain, or dysmenorrhea, and impairment of physical functioning is not only associated with social isolation and withdrawal, but it is also associated with poorer social functioning (e.g. less support and more strain; MacGregor et al., 2023; Gagnon et al., 2023). Indeed, menstrual pain has been notoriously misunderstood and mistreated by health professionals and the broader community (Ballard et al., 2006; De Graaff et al., 2013; Missmer et al., 2022), resulting in women feeling ignored and disbelieved (Culley et al., 2013; Young et al., 2016). Future research would benefit in determining what type of social connections women with more severe pain and impairment of physical functioning need to mitigate any loneliness experiences.

Contrary to our expectations, pain severity was not directly associated with loneliness. This is inconsistent with research that found higher levels of pain severity were associated with higher levels of loneliness in older adults with orofacial pain (Boggero et al., 2019) and research that found pain severity was an early indicator of loneliness in adults with inflammatory pain conditions (Loeffler & Steptoe, 2021). However, these studies did not include both pain severity and impairment of physical functioning as predictors. Given that we observed that pain severity is an indirect indicator of higher loneliness by decreasing physical functioning over time, it seems that pain severity contributes most strongly to loneliness when menstrual pain impairs physical function since reduced physical function can subsequently impair social, recreational and vocational activities. These findings concur with recent qualitative work where women with endometriosis described the loss of liberty, autonomy and social connectedness attributed to their dysmenorrhea. In particular, while participants expressed difficulties in performing their usual familial, work and social activities even when dysmenorrhea was mild or moderate in severity, these themes were more commonly reported in women with more severe pain (Allyn et al., 2020; Peterson et al., 2023). Our study extends from these findings by statistically modelling within‐person change over time, allowing for a more precise understanding of how physical impairment contributes to loneliness. As such, it would be worthwhile to track women with high menstrual pain severity in intervention efforts to ensure that support is provided when the pain begins to lead to impairment of physical functioning.

Although our study is the first to quantitatively examine pain factors and loneliness in a sample of women with menstrual pain and used a prospective study design and controlled for important confounders, it is not without limitations. First, the sample in the current study reported average levels of loneliness overall. Participants were primarily recruited online via dysmenorrhea peer support groups. Since providing and receiving peer support is known to mitigate loneliness (Cacioppo et al., 2015), our participants may characteristically express lower average loneliness as a function of their involvement with these groups than others with dysmenorrhea might. Future samples should target women experiencing social isolation (e.g. unmarried, living alone, not receiving peer support; Naito et al., 2021). Related to this, it is possible that this study did not include women with the most severe dysmenorrhea experiences. This study was also limited by its inclusion/exclusion criteria, as only women were included, thus excluding people who were assigned female at birth but do not identify as women. Given that there is evidence to suggest that transgender people are more vulnerable to experiencing loneliness compared with cisgender people (Hajek et al., 2023), and 43% of transgender people utilizing gender‐affirming health care report dysmenorrhea symptoms (Ferrando et al., 2021), this may limit the generalizability of our findings from an important cohort of people with dysmenorrhea. Methodologically, while our model temptingly alludes to a causal pathway of pain to physical function to loneliness, it is important to note that the timing of the measurements over multiple years allows for historical effects, such as the COVID‐19 pandemic that cannot be easily accounted for or controlled. Future research should consider ecological momentary assessment or temporal network analysis to better capture individual‐level change with greater precision. For example, future time‐intensive designs allowing for within‐person associations might benefit by exploring the ways in which other variables interact to contribute to loneliness. We also suggest that the incorporation of qualitative data would allow researchers to better understand the experiences of individual women and how well these are captured in sample or population‐level analyses. Likewise, research that focuses on younger people could guide prevention efforts over longitudinal timeframes, such as the transition from adolescence into young adulthood. Finally, we only assessed loneliness at Time‐3, so we could not examine the expected bidirectional associations between menstrual pain severity and physical functioning and loneliness. Loneliness likely exacerbates pain severity and physical functioning (cf. Emerson et al., 2018; Mou et al., 2019; Powell et al., 2022). We intend to address these limitations in future work.

### Conclusion

This study adds to the growing body of research on the impacts of menstrual pain by examining the associations between menstrual pain severity, physical functioning and loneliness over time in a community cohort. Poorer physical functioning was associated with greater loneliness, even when controlling for important confounds. Contrary to our expectations, menstrual pain severity was not directly associated with loneliness. Instead, it was indirectly associated with greater loneliness via poorer physical functioning. These findings suggest that menstrual pain alone is insufficient to drive loneliness except for where the pain contributes to reduced physical functioning, and as such, clinicians could work to more specifically identify pain that interferes with functioning, regardless of severity. In particular, clinicians should discuss loneliness and other social contributors to pain with their patients.

## AUTHOR CONTRIBUTIONS

**J. McCurry:** Conceptualization; methodology; writing – original draft. **D. Skvarc:** Methodology; data curation; formal analysis; writing – original draft; conceptualization. **S. Evans:** Conceptualization; data curation; writing – review and editing; project administration. **A. Mikocka‐Walus:** Supervision; investigation; writing – review and editing. **M. L. Druitt:** Validation; investigation; writing – review and editing. **L. Payne:** Validation; investigation; writing – review and editing. **E. M. Marshall:** Conceptualization; methodology; data curation; writing – original draft; validation.

## CONFLICT OF INTEREST STATEMENT

The authors have no conflicts of interest to report.

## ETHICS STATEMENT

Ethics approval was obtained from Deakin University. Due to university ethics requirements, the data are only available by directly contacting the corresponding author. The study design and analyses were not pre‐registered.

## Supporting information


Data S1.

## Data Availability

The data that support the findings of this study are available on request from the corresponding author. The data are not publicly available due to privacy or ethical restrictions.
