# Supplementary material for: In pain and lonely? A longitudinal study examining the associations between menstrual pain, physical functioning and loneliness
Source: Br J Health Psychol. 2025 May 26;30(3):e12805. doi: 10.1111/bjhp.12805 (PMC12104796; doi:10.1111/bjhp.12805)
Supplement: Supplementary file 1 — Data S1. [file BJHP-30-0-s001.docx]

**Model Coefficients**

| Regression Weights |  |  |  |  |  |  |  |  |
| --- | --- | --- | --- | --- | --- | --- | --- | --- |
|  |  |  |  |  |  |  |  |  |
| Endogenous |  | Exogenous | Estimate | Beta | S.E. | C.R. | P | Label |
| Pain2 | <--- | Pain1 | 0.19 | 0.182 | 0.098 | 1.928 | 0.054 | d |
| Qol2 | <--- | Qol1 | 0.465 | 0.442 | 0.085 | 5.442 | *** | e |
| Qol2 | <--- | Pain1 | -0.618 | -0.216 | 0.225 | -2.752 | 0.006 |  |
| Pain2 | <--- | Qol1 | -0.135 | -0.353 | 0.033 | -4.096 | *** |  |
| Pain3 | <--- | Pain2 | 0.19 | 0.202 | 0.098 | 1.928 | 0.054 | d |
| Qol3 | <--- | Qol2 | 0.465 | 0.463 | 0.085 | 5.442 | *** | e |
| Qol3 | <--- | Pain2 | -0.576 | -0.209 | 0.208 | -2.776 | 0.005 |  |
| Pain3 | <--- | Qol2 | -0.113 | -0.329 | 0.034 | -3.279 | 0.001 |  |
| T1_PAIN_LEVEL | <--- | Pain1 | 1 | 0.669 |  |  |  |  |
| T2_PAIN_LEVEL | <--- | Pain2 | 1 | 0.685 |  |  |  |  |
| T3_PAIN_LEVEL | <--- | Pain3 | 1 | 0.663 |  |  |  |  |
| T1_WHO_QoL_D1 | <--- | Qol1 | 1 | 0.791 |  |  |  |  |
| T2_WHO_QoL_D1 | <--- | Qol2 | 1 | 0.805 |  |  |  |  |
| T3_WHO_QoL_D1 | <--- | Qol3 | 1 | 0.807 |  |  |  |  |
| T1_PAIN_LEVEL | <--- | StablePain | 1 | 0.743 |  |  |  |  |
| T2_PAIN_LEVEL | <--- | StablePain | 1 | 0.728 |  |  |  |  |
| T3_PAIN_LEVEL | <--- | StablePain | 1 | 0.749 |  |  |  |  |
| T1_WHO_QoL_D1 | <--- | StableQoL | 1 | 0.612 |  |  |  |  |
| T2_WHO_QoL_D1 | <--- | StableQoL | 1 | 0.593 |  |  |  |  |
| T3_WHO_QoL_D1 | <--- | StableQoL | 1 | 0.591 |  |  |  |  |
| T3_UCLA_LONE_TOT | <--- | Pain3 | 0.056 | 0.027 | 0.166 | 0.338 | 0.735 |  |
| T3_UCLA_LONE_TOT | <--- | Qol3 | -0.267 | -0.371 | 0.058 | -4.562 | *** |  |
| T3_UCLA_LONE_TOT | <--- | T1_ENDO | 0.37 | 0.058 | 0.358 | 1.035 | 0.301 |  |
| T3_UCLA_LONE_TOT | <--- | T1_CHRONIC_ILLNESSES | -1.007 | -0.157 | 0.359 | -2.803 | 0.005 |  |
| T3_UCLA_LONE_TOT | <--- | T1_EMPLOYMENT_STATUS | 0.131 | 0.108 | 0.068 | 1.93 | 0.054 |  |
| T3_UCLA_LONE_TOT | <--- | AgeYears | -0.039 | -0.099 | 0.028 | -1.412 | 0.158 |  |
| T3_UCLA_LONE_TOT | <--- | T1_MARITAL_STATUS | 0.138 | 0.039 | 0.212 | 0.648 | 0.517 |  |
| T3_UCLA_LONE_TOT | <--- | T1_CHILDREN | -0.103 | -0.015 | 0.474 | -0.216 | 0.829 |  |
|  |  |  |  |  |  |  |  |  |
| Intercepts: (Group number 1 - Default model) |  |  |  |  |  |  |  |  |
|  |  |  |  |  |  |  |  |  |
|  |  |  | Estimate | S.E. | C.R. | P | Label |  |
| T1_PAIN_LEVEL |  |  | 6.52 | 0.049 | 132.092 | *** |  |  |
| T2_PAIN_LEVEL |  |  | 6.179 | 0.078 | 79.684 | *** |  |  |
| T3_PAIN_LEVEL |  |  | 6.043 | 0.092 | 65.911 | *** |  |  |
| T3_UCLA_LONE_TOT |  |  | 9.423 | 1.799 | 5.239 | *** |  |  |
| T1_WHO_QoL_D1 |  |  | 23.153 | 0.126 | 183.253 | *** |  |  |
| T2_WHO_QoL_D1 |  |  | 23.813 | 0.188 | 126.367 | *** |  |  |
| T3_WHO_QoL_D1 |  |  | 23.244 | 0.235 | 98.907 | *** |  |  |
|  |  |  |  |  |  |  |  |  |
| Covariances: (Group number 1 - Default model) |  |  |  |  |  |  |  |  |
|  |  |  |  |  |  |  |  |  |
|  |  |  | Covariance | Correlation | S.E. | C.R. | P | Label |
| StableQoL | <--> | StablePain | -1.277 | -0.248 | 0.831 | -1.537 | 0.124 |  |
| Pain1 | <--> | Qol1 | -3.187 | -0.533 | 0.822 | -3.876 | *** |  |
| u1 | <--> | v1 | -1.765 | -0.377 | 0.346 | -5.098 | *** |  |
| u2 | <--> | v2 | -1.441 | -0.329 | 0.33 | -4.362 | *** |  |
| T1_ENDO | <--> | T1_CHRONIC_ILLNESSES | 0.05 | 0.218 | 0.005 | 9.414 | *** |  |
| T1_CHRONIC_ILLNESSES | <--> | T1_EMPLOYMENT_STATUS | -0.052 | -0.043 | 0.027 | -1.907 | 0.057 |  |
| T1_ENDO | <--> | T1_EMPLOYMENT_STATUS | 0.106 | 0.086 | 0.027 | 3.859 | *** |  |
| T1_ENDO | <--> | StablePain | -0.428 | -0.539 | 0.025 | -17.092 | *** |  |
| T1_CHRONIC_ILLNESSES | <--> | StablePain | -0.1 | -0.128 | 0.023 | -4.353 | *** |  |
| T1_EMPLOYMENT_STATUS | <--> | StablePain | -0.133 | -0.032 | 0.122 | -1.092 | 0.275 |  |
| T1_EMPLOYMENT_STATUS | <--> | StableQoL | -1.806 | -0.227 | 0.312 | -5.78 | *** |  |
| T1_CHRONIC_ILLNESSES | <--> | StableQoL | 0.631 | 0.423 | 0.06 | 10.516 | *** |  |
| T1_ENDO | <--> | StableQoL | 0.768 | 0.508 | 0.061 | 12.512 | *** |  |
| T1_EMPLOYMENT_STATUS | <--> | AgeYears | -2.582 | -0.13 | 0.432 | -5.975 | *** |  |
| T1_CHRONIC_ILLNESSES | <--> | AgeYears | -0.365 | -0.098 | 0.069 | -5.274 | *** |  |
| T1_ENDO | <--> | AgeYears | -0.571 | -0.152 | 0.084 | -6.787 | *** |  |
| AgeYears | <--> | T1_MARITAL_STATUS | 2.629 | 0.389 | 0.155 | 16.949 | *** |  |
| T1_EMPLOYMENT_STATUS | <--> | T1_MARITAL_STATUS | -0.146 | -0.066 | 0.048 | -3.043 | 0.002 |  |
| T1_CHRONIC_ILLNESSES | <--> | T1_MARITAL_STATUS | -0.015 | -0.037 | 0.009 | -1.757 | 0.079 |  |
| T1_ENDO | <--> | T1_MARITAL_STATUS | -0.05 | -0.119 | 0.009 | -5.297 | *** |  |
| T1_MARITAL_STATUS | <--> | StablePain | -0.028 | -0.02 | 0.042 | -0.67 | 0.503 |  |
| T1_MARITAL_STATUS | <--> | StableQoL | -0.226 | -0.083 | 0.105 | -2.143 | 0.032 |  |
| AgeYears | <--> | StableQoL | -1.42 | -0.058 | 0.941 | -1.509 | 0.131 |  |
| AgeYears | <--> | StablePain | -1.37 | -0.107 | 0.374 | -3.661 | *** |  |
| T1_MARITAL_STATUS | <--> | T1_CHILDREN | -0.143 | -0.368 | 0.009 | -16.008 | *** |  |
| AgeYears | <--> | T1_CHILDREN | -2.002 | -0.574 | 0.085 | -23.593 | *** |  |
| T1_EMPLOYMENT_STATUS | <--> | T1_CHILDREN | -0.103 | -0.09 | 0.025 | -4.156 | *** |  |
| T1_ENDO | <--> | T1_CHILDREN | 0.006 | 0.028 | 0.005 | 1.298 | 0.194 |  |
| T1_CHILDREN | <--> | StablePain | 0.13 | 0.176 | 0.022 | 6.025 | *** |  |
| T1_CHILDREN | <--> | StableQoL | 0.066 | 0.047 | 0.053 | 1.249 | 0.212 |  |
|  |  |  |  |  |  |  |  |  |
|  |  |  |  |  |  |  |  |  |
|  |  |  | Estimate | S.E. | C.R. | P | Label |  |
| Pain1 |  |  | 2.195 | 0.327 | 6.707 | *** |  |  |
| Qol1 |  |  | 16.308 | 2.73 | 5.974 | *** |  |  |
| StablePain |  |  | 2.705 | 0.342 | 7.916 | *** |  |  |
| StableQoL |  |  | 9.787 | 2.733 | 3.581 | *** |  |  |
| u1 |  |  | 1.852 | 0.218 | 8.512 | *** |  |  |
| v1 |  |  | 11.83 | 1.024 | 11.558 | *** |  |  |
| u2 |  |  | 1.651 | 0.179 | 9.227 | *** |  |  |
| v2 |  |  | 11.588 | 1.126 | 10.296 | *** |  |  |
| T1_ENDO |  |  | 0.233 | 0.007 | 31.668 | *** |  |  |
| T1_CHRONIC_ILLNESSES |  |  | 0.227 | 0.007 | 31.237 | *** |  |  |
| T1_EMPLOYMENT_STATUS |  |  | 6.469 | 0.198 | 32.651 | *** |  |  |
| AgeYears |  |  | 60.775 | 1.77 | 34.34 | *** |  |  |
| T1_MARITAL_STATUS |  |  | 0.753 | 0.023 | 32.689 | *** |  |  |
| T1_CHILDREN |  |  | 0.2 | 0.006 | 32.813 | *** |  |  |
| e7 |  |  | 7.564 | 0.675 | 11.213 | *** |  |  |
|  |  |  |  |  |  |  |  |  |
| R2 |  |  |  |  |  |  |  |  |
|  |  |  |  |  |  |  |  |  |
|  |  |  | Estimate |  |  |  |  |  |
| Qol2 |  |  | 0.344 |  |  |  |  |  |
| Pain2 |  |  | 0.226 |  |  |  |  |  |
| Qol3 |  |  | 0.364 |  |  |  |  |  |
| Pain3 |  |  | 0.222 |  |  |  |  |  |
| T3_UCLA_LONE_TOT |  |  | 0.195 |  |  |  |  |  |

**Dropout Analysis**
